# PETRA: a pedestal exoskeletal testbed with real-time architecture for upper limb rehabilitation robotics

**DOI:** 10.3389/fresc.2026.1859475

**Published:** 2026-07-03

**Authors:** Andres Garcia, Kevin Fernández, Andres Nenger, Redhwan Algabri, Carlos Salazar, Jonathan Leon, Francisco Yumbla

**Affiliations:** 1 Facultad de Ingeniería en Mecánica y Ciencias de la Producción, Escuela Superior Politecnica del Litoral, ESPOL, Guayaquil, Ecuador; 2 Department of Computer Science and Engineering, Sejong University, Seoul, Republic of Korea; 3 Facultad de Ingeniería en Electricidad y Computación, Escuela Superior Politecnica del Litoral, ESPOL, Guayaquil, Ecuador

**Keywords:** additive manufacturing, exoskeletal platform, exoskeletal testbed, open-source, rehabilitation robotics, trapezoidal motion

## Abstract

More than 85% of stroke survivors present motor impairment in the upper limb, yet access to rehabilitation robotics remains concentrated in high-income countries due to costs exceeding 10,000 USD and architectures dependent on external computers. This economic barrier limits the ability of researchers in low- and middle-income countries (LMICs) to develop and validate new therapeutic strategies. This article presents PETRA (Pedestal Exoskeletal Testbed with Real-time Architecture), an open-source, 3-degree-of-freedom kinematic validation testbed manufactured in PETG for upper limb rehabilitation research at a cost under 25 USD per joint. PETRA adopts a fixed pedestal configuration that decouples the robot’s dynamics from the user’s biomechanical disturbances, providing a controlled environment for characterizing parametric cycloidal transmissions and deterministic dual-core firmware without PC/ROS dependency. Experimental validation with over 300 trials confirms positioning accuracies within clinical requirements while identifying polymer friction as the primary technical constraint. By providing a low-cost, replicable methodological standard, PETRA facilitates the transition from conceptual design to robust assistive technology in institutions with limited resources.

## 1 Introduction

Stroke constitutes a global public health crisis with devastating consequences for upper limb motor function. According to the Global Burden of Disease Study 2021, stroke is the second leading cause of death worldwide, responsible for approximately 7.3 million annual deaths (10.7% of total mortality), and the third leading cause of combined disability, with more than 160 million disability-adjusted life years (DALYs) (1). In 2021, there were an estimated 93.8 million prevalent cases and 11.9 million incident cases, with an increase of 70% in incidence and 86% in prevalence compared to 1990 (1, 2). Of the total survivors, between 55% and 75% present motor dysfunction, and upper limb involvement reaches up to 85% of cases (3), with arm weakness being the most frequent sequel and the one with the slowest recovery compared to the lower limb (4, 5). Approximately 30% of patients do not recover fine control of the upper extremity, compromising daily activities such as eating or dressing (3). The global cost of stroke exceeds 890 billion dollars annually (0.66% of global GDP), with a projection to double by 2050 (2), and 87% of deaths and 89% of DALYs are concentrated in low- and middle-income countries (LMICs) (1, 2).

In Latin America and the Caribbean (LAC), the epidemiological transition toward older urban populations has increased the prevalence of cardiovascular risk factors, positioning stroke as the primary cause of death in most countries in the region (6). LAC records the fourth highest stroke burden worldwide, with 6.8 million DALYs in 2019, predominantly attributable to premature deaths (89.5% of DALYs) (7). The pooled prevalence of stroke in the region is estimated at 32 per 1,000 inhabitants, with a crude annual incidence of first event of approximately 119 per 100,000 people, and a one-month case fatality rate of 21.1% (8, 9). Up to 90% of this burden would be reducible through the control of systolic arterial hypertension (53%) and body mass index (37%), factors that are highly preventable but insufficiently controlled in the region (7). Despite these alarming indicators, studies characterizing the stroke burden in LAC are scarce, and access to specialized rehabilitation services remains fragmented and inequitable (6, 10).

In Ecuador, cerebrovascular disease has remained among the leading causes of mortality since 1990 (11). A 17-year analysis (2001–2017) of hospital records from the National Institute of Statistics and Censuses (INEC) revealed significant differences in stroke incidence and mortality according to geographic region and altitude of residence (12). More recent studies based on hospital discharges for the 2015–2020 period document ethno-demographic disparities in the disease burden, with variations in DALYs attributable to stroke reflecting differential access to health services according to ethnic group and socioeconomic level (13). Cardiovascular mortality, including cerebrovascular, experienced an additional increase during the COVID-19 pandemic, representing 30.4% of the excess mortality recorded in 2020 (14). These figures evidence the urgent need for accessible and replicable rehabilitation solutions that can be deployed in clinical and academic settings with limited resources.

Rehabilitation robotics has emerged as a promising tool to complement conventional therapy, offering high-repeatability movement assistance that promotes neuroplasticity through motor relearning protocols (15, 16). However, commercial medical exoskeletons present costs ranging between 10,000 and more than 100,000 USD per unit (17, 18), an economic barrier that restricts their adoption in LMICs where the largest proportion of the global stroke burden resides. Upper limb robotic rehabilitation systems are particularly valued for their ability to replicate the physiological joint movements of the shoulder and elbow, the joints with the greatest documented clinical impact on functional recovery after a stroke (15, 19). Nevertheless, widespread clinical adoption remains limited by high acquisition and maintenance costs, operational complexity, the need for trained personnel, and restricted accessibility in regions with deficient health infrastructure (17, 20). The need for affordable, robust, and open-source platforms are, therefore, a constant challenge for researchers and therapists seeking to bring the benefits of robot-assisted rehabilitation beyond privileged laboratories.

Recent literature offers various robotic platforms oriented towards both rehabilitation and education (21, 22). OpenExo (21) proposes a completely open-source modular exoskeleton framework, but depends on a computational architecture based on a Teensy microcontroller with BLE communication and requires high-cost commercial actuators. EULR (23), a 3D-printed exoskeleton with ESP32 microcontrollers and force sensors, manages to reduce costs to 98.4 USD per unit, but focuses exclusively on hand rehabilitation and does not address the proximal joints of the shoulder and elbow. Platforms such as POWERUP (22) offer passive assistance through elastic bands but lack active actuation and closed-loop control. On the other hand, systems that do integrate active actuation with multiple degrees of freedom depend on complex software stacks like ROS (24), increasing the technical barrier to entry. Additionally, mechanical transmissions based on conventional gear trains introduce non-negligible backlash that degrades positioning repeatability, a critical parameter in rehabilitation protocols where angular fidelity directly determines therapeutic efficacy and efficiency (25).

Despite these advances, there is a significant gap in the availability of platforms designed as controlled research environments for kinematic characterization. To address this gap, this article presents PETRA (Pedestal Exoskeletal Testbed with Real-time Architecture), an open-source, 3-DOF kinematic validation testbed. PETRA is designed as a methodological standard that decouples robotic dynamics from human biomechanical disturbances, allowing for the rigorous evaluation of embedded control strategies and low-cost transmissions in a laboratory setting before their integration into wearable assistive systems.

The rest of this article is organized as follows. Section 2 presents the clinical and methodological motivation that informs the design of PETRA, including the kinematic analysis of the target joints and the definition of safety limits. Section 3 describes the mechanical design of the prototype, including structural geometry, cycloidal transmissions, and the manufacturing process. Section 4.1 details the electronic architecture and signal conditioning of the custom PCB. Section 4.2 presents the dual-core firmware architecture and the embedded trapezoidal motion planner. Section 5 reports the results of the experimental validation, including the characterization of positioning accuracy across the three axes. Finally, Section 6 summarizes the main findings and proposes directions for future work. All design files, firmware source code, and documentation are publicly available in the project’s GitHub repository link^1^.

## 2 Conceptual system

The transition from laboratory prototypes to clinical rehabilitation systems is frequently hindered by the lack of controlled testing environments (26). While most current developments focus on wearability, PETRA prioritizes methodological standardization. By adopting a fixed pedestal configuration, a reference frame is established where the robot’s dynamics are independent of the user’s biomechanical disturbances, such as joint misalignments or variations in harness tension. This isolation is fundamental to validate the determinism of the dual-core architecture and the accuracy of the cycloidal transmissions under constant load conditions, allowing PETRA to function as a reference testbed for the evaluation of anthropomorphic control algorithms before their implementation in mobile systems.

The human arm constitutes a complex kinematic chain that facilitates the placement of the hand in three-dimensional space (25). This chain presents unique engineering challenges, as it requires a balance between range of motion and structural stability. For the design of PETRA, the analysis focuses on two fundamental joints with direct clinical relevance in motor rehabilitation protocols (15). In the context of recovery after a stroke, the restoration of proximal mobility (shoulder and elbow) is a critical prerequisite for recovering the distal functionality of the hand. For this reason, the platform emulates the flexion-extension and abduction/adduction axes using human proportions to ensure a clinically representative spherical workspace. This approach allows for the validation of the actuators’ response to gravitational load variations that occur during motor relearning protocols, establishing a robust testing standard before integration into active assistance systems.

**Figure 1:**
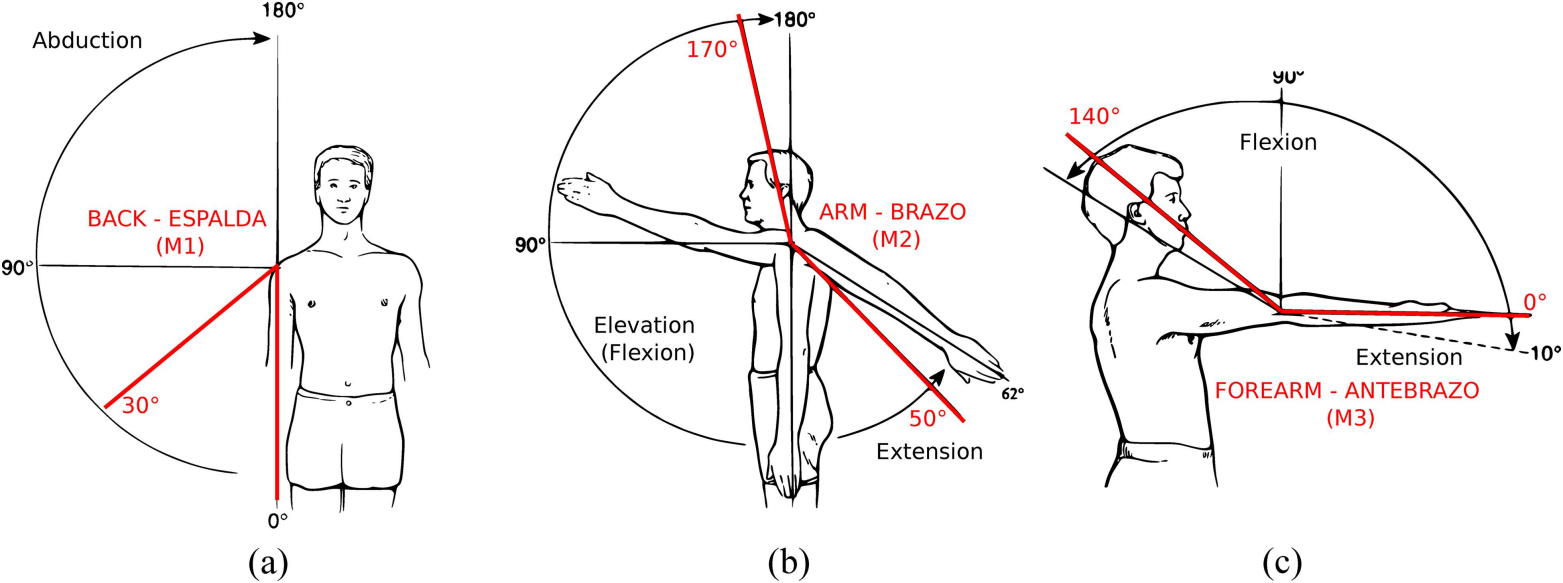
Diagram of maximum anatomical ranges of motion (ROM) compared with the limits configured in PETRA (marked in red). **(a)** shoulder adduction and abduction, **(b)** frontal elevation and extension, **(c)** elbow flexion-extension.

**The glenohumeral joint** is defined as a spherical type system with three physiological degrees of freedom, allowing the execution of flexion/extension, abduction/adduction, and internal/external rotation movements (27). However, in order to establish a testing standard that prioritizes mechanical stability and efficient torque delivery, the analysis for PETRA focuses on the two movements with the greatest documented clinical impact on functional recovery: abduction/adduction and frontal elevation/extension. These planes of motion, whose maximum anatomical ranges are compared with the proposed operational limits in Figures 1a,b, are conceptually emulated through a configuration of two perpendicular revolute joints. This kinematic simplification allows for the validation of control in the proximal axes most critical for neuroplasticity without introducing the mechanical instabilities of a full spherical system at this stage of research.

**The humero-ulnar joint** mechanically acts as a hinge or pure revolute joint, whose primary function is elbow flexion-extension, facilitating the variation of the functional distance between the shoulder and the hand for reaching tasks (27). In the PETRA architecture, this movement is proposed as a third degree of freedom (DoF) of revolute type, oriented to cover the therapeutic range illustrated in Figure 1c. The inclusion of this axis in the model responds to the need to evaluate how control algorithms manage inertia variations and the load moments resulting from the displacement of the forearm segment. By maintaining human proportions in this conceptual design, it is ensured that the system’s workspace is representative for anthropomorphic and biomechanical research, allowing a direct transfer of torque and precision results toward real clinical protocols.

This resulting 3-degree-of-freedom (DoF) configuration (composed of two axes in the shoulder complex and one in the elbow) provides an optimal balance between system complexity and the coverage of movement archetypes with the greatest functional impact on functional motor rehabilitation. By prioritizing this kinematic simplification, PETRA not only reduces the computational load of the control but also facilitates rapid calibration and adjustment processes, which are critical requirements for a platform oriented toward repetitive experimentation and the validation of neuroplasticity protocols.

To ensure a safe research environment, the system’s operational limits have been defined by integrating redundant safety margins of $10^{\circ }$ for abduction and elbow flexion-extension movements (as illustrated in Figures 1b,c), along with an operational margin of $150^{\circ }$ for frontal elevation (Figure 1a). These offsets, implemented directly in the firmware logic, act as an essential biomechanical protection layer that actively prevents the actuators from forcing the mechanical joints beyond the patient’s safe anatomical ranges. In this way, PETRA is established as a robust testing standard that combines the fidelity of human movement with the safety rigor necessary for assistive research.

## 3 Prototype design

**Figure 2:**
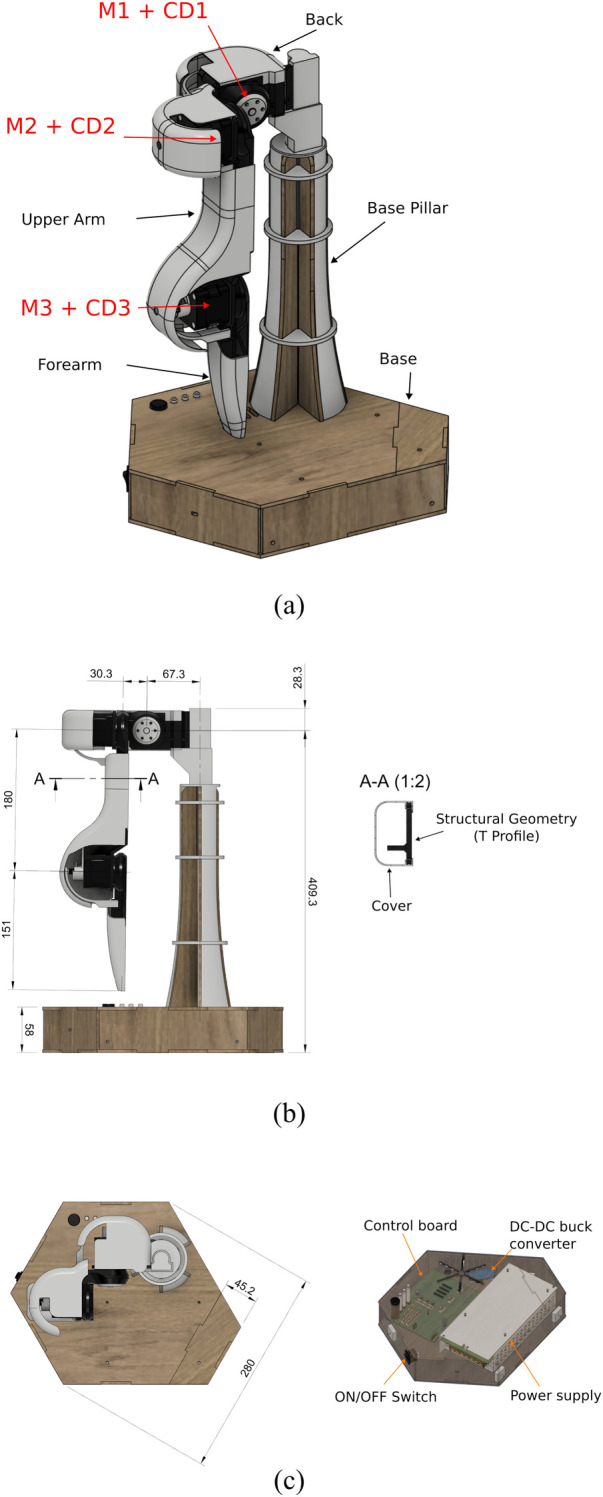
Structural architecture and engineering details of the PETRA prototype: **(a)** Isometric view with the location of actuators and cycloidal (CD) transmissions, **(b)** Anthropometric dimensioning and T-slot profile details, **(c)** Hexagonal base geometry for optimization of the support polygon, view of the integrated internal compartment housing the 24 V power supply, *buck* converter, and custom control board, configuring the pedestal as an autonomous and transportable test station.

The design of PETRA focused on creating a platform that combines structural lightness with superior mechanical rigidity. The system was manufactured entirely through additive manufacturing (FDM) using PETG, whose selection is based on its ability to withstand the cyclic stresses of rehabilitation without the fragility of PLA or the thermal deformations of ABS, while maintaining comparable cost and accessibility (28). As illustrated in Figure 2, the segment lengths of 180 mm and 151 mm are not arbitrary, but rather respond to the human proportions necessary to ensure that the workspace and load moments are clinically representative. This dimensional consistency is fundamental to meeting the platform’s objective: providing a testing environment where the actuators’ response is directly transferable to a real assistance scenario, eliminating ”confounding variables” derived from poor anthropometric adaptation.

The geometry of the segments employs T-profiles (Figure 2b) to optimize the second moment of area, allowing the structure to withstand the dynamic loads of the motors with minimal flexion without increasing the system’s inertia. However, the greatest innovation in PETRA’s architecture lies in its configuration as an autonomous and transportable testing station. The hexagonal base (Figure 2) not only ensures operational stability by maximizing the support polygon and ensuring that the center of mass (CoM) always remains within the support area, but also acts as an integrated chassis. As observed in the detail views of Figure 2, the base internally houses the 24 V power supply, a DC-DC buck converter, and the custom control board. This hidden integration of heavy components shifts the center of gravity to the lowest point of the pedestal, reducing structural vibrations and allowing the testbed to be a compact unit, easy to move between laboratories and ready to operate without depending on external infrastructure, thus reinforcing its role as a rapid-deployment research standard.

**Figure 3:**
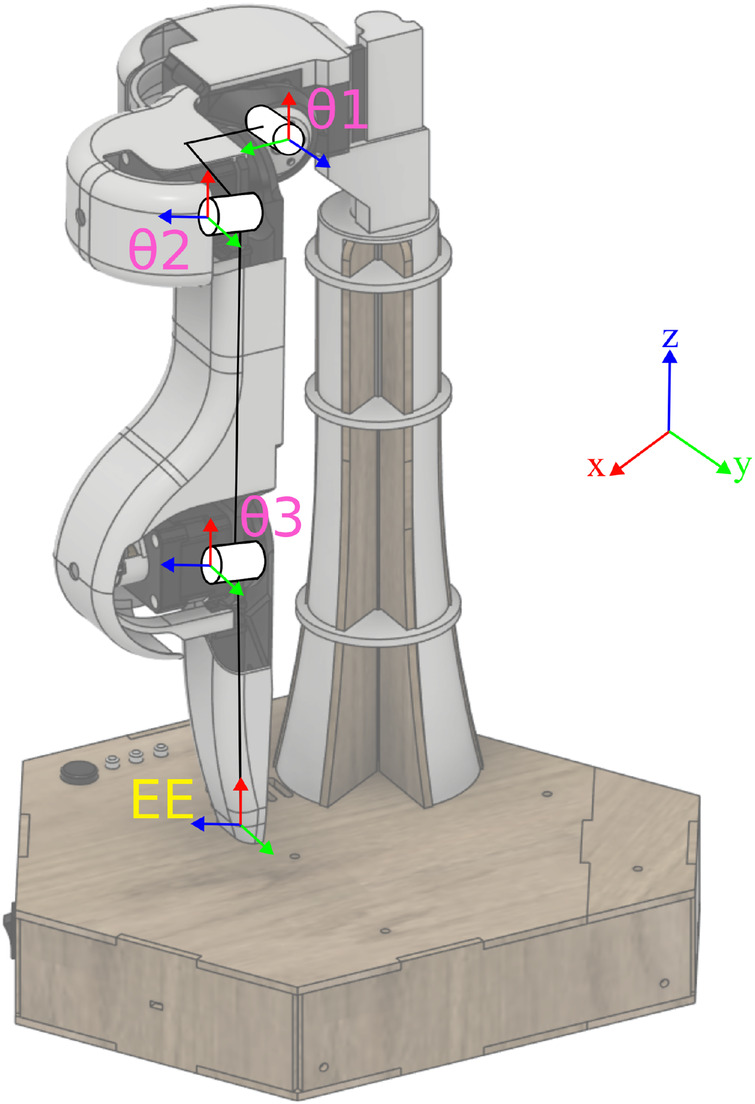
Diagram of the PETRA joint configuration and coordinate frames, identifying the rotation axes ($\theta_{1},\theta_{2},\theta_{3}$) and the end-effector (EE) location.

**Figure 4:**
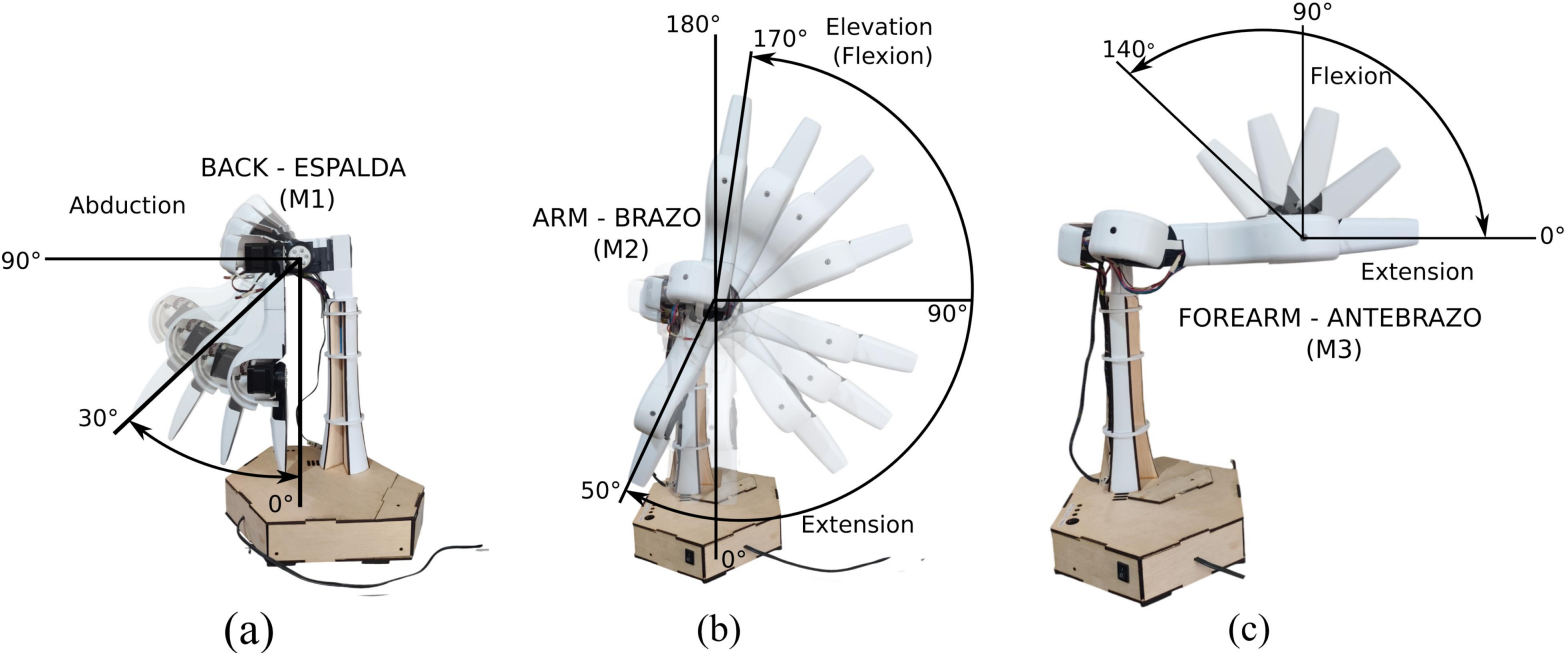
Configuration of the PETRA workspace replicating anatomical ROM through multi-pose physical validation: **(a)** shoulder abduction, **(b)** frontal elevation and extension, and **(c)** elbow flexion-extension. The superimposed frames demonstrate the robot’s capacity to cover the full therapeutic arcs required for upper limb rehabilitation.

As a result of the kinematic strategy established in Section 2, Figure 3 details the physical implementation of the rotation axes ($\theta_{1},\theta_{2},\theta_{3}$) and the location of the end effector (EE). This architecture allows the translation of the conceptual model of anatomical emulation into a deterministic coordinate system, facilitating the execution of precise spatial trajectories. The resulting workspace (Workspace) validation is presented in Figure 4, where the critical poses that the robot is capable of reaching during a rehabilitation protocol are illustrated. In these views, it is verified that the platform covers the motion arcs necessary for functional reaching and elevation tasks, strictly respecting the safety *offsets* configured in the *firmware*. This correspondence between the theoretical design and the physical reach ensures that PETRA functions as a high-fidelity *testbed*, allowing the validation of control algorithms in a spherical workspace that is clinically representative and mechanically stable.

To address the requirements of high torque density and the elimination of mechanical play (backlash) in each joint, a parametric cycloidal reducer (CD1, CD2, and CD3) was designed and manufactured, compactly integrated into the structure (Figure 2a). The disk profile design was generated using the CycloidalDrive plugin in Fusion 360 (29), optimizing the epitrochoid geometry so that multiple lobes remain in simultaneous contact with the ring of fixed pins at all times. This feature allows approximately one-third of the lobes to distribute the load concurrently, resulting in a high torque density significantly superior to that of conventional gears. This distribution of forces ensures a motion transmission without play, which is a fundamental advantage over standard gear trains in motor rehabilitation applications where direction reversals must be smooth and precise to ensure user comfort and kinematic data integrity (25, 29).

**Figure 5:**
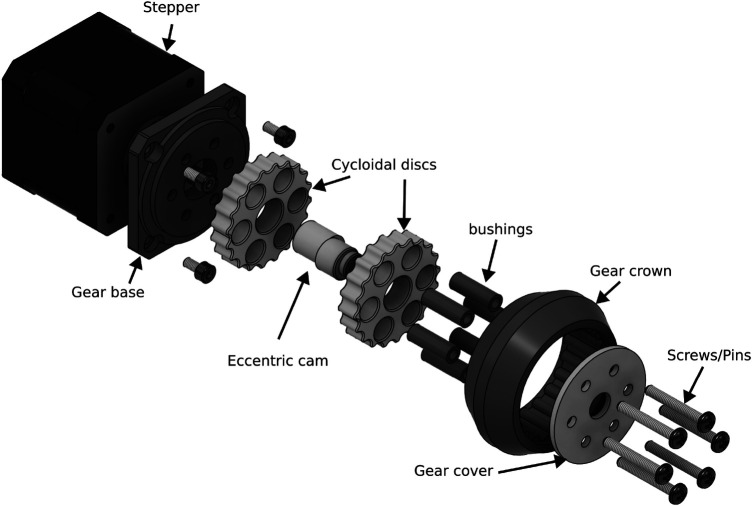
Exploded view of the cycloidal transmission: stepper motor, eccentric shaft, cycloidal disks $180^{\circ }$ out of phase, reaction pins with bushings, ring gear, and output cover.

As detailed in the exploded view of Figure 5, each transmission unit employs an architecture of two cycloidal disks phased $180^{\circ }$ apart from each other to cancel the eccentric vibration generated by the input cam and ensure concentric operation. Due to the use of FDM-manufactured polymers, the cycloidal geometry offers critical structural robustness: the wide base of the lobes better resists shear stresses compared to the thin teeth of an involute gear, which typically fail due to layer separation in printed parts. To mitigate the mechanical degradation of the material, the disks interact with six 1/8-inch reaction pins equipped with plastic bushings that reduce direct wear on the PETG surfaces. The reducer housing couples directly to the faceplate of the NEMA 17 stepper motor (30) using four M3 screws, resulting in a compact and modular assembly whose key design parameters are presented in Table 1.

**Table 1:** Cycloidal drive design parameters.

Parameter	Value
Reduction ratio	20:1
Eccentric amount	0.75 mm
Ring pin diameter	3.5 mm
Pin pitch diameter	36 mm
Output pins	6
Output pin diameter	5.3 mm
Center-to-pin distance	22 mm
Min. pressure angle	24.88 deg
Output speed	15 RPM
Material	PETG

The fundamental design parameters governing the performance of this transmission are presented in Table 1. The 20:1 reduction ratio was selected to limit the output speed to a maximum of 15 RPM, a cadence that faithfully replicates the typical rhythm of human therapeutic movement (27). This reduction multiplies the actuator’s native resolution to reach 4,000 steps per revolution, allowing for fine-grained position control and a smooth dynamic response managed by the trapezoidal velocity profile embedded in the firmware. Finally, it should be noted that the absence of metallic bearings at the PETG interfaces introduces friction losses that reduce the available torque at the output shaft relative to the theoretical value; its experimental characterization and quantification are discussed in depth in Section 5.

## 4 System architecture

For PETRA to function as an autonomous testing station, its architecture integrates electronic design with real-time control. This section details the two technical pillars that enable the robot’s stability: a custom PCB focused on power management and analog signal integrity, and a dual-core firmware designed to isolate critical motor actuation from wireless communication services. By unifying these components, the system eliminates the dependency on external computers and ensures a dynamic response that complies with the safety operational ranges established for rehabilitation.

### 4.1 Electronics and signal

Following the functional validation of the electronic schematic on a breadboard, a single-layer printed circuit board (PCB) was designed using KiCad. The transition to a dedicated board design allowed for the elimination of wiring instabilities and variations in contact resistance observed in the initial phases, establishing a robust platform for the dynamic control of the system.

**Figure 6:**
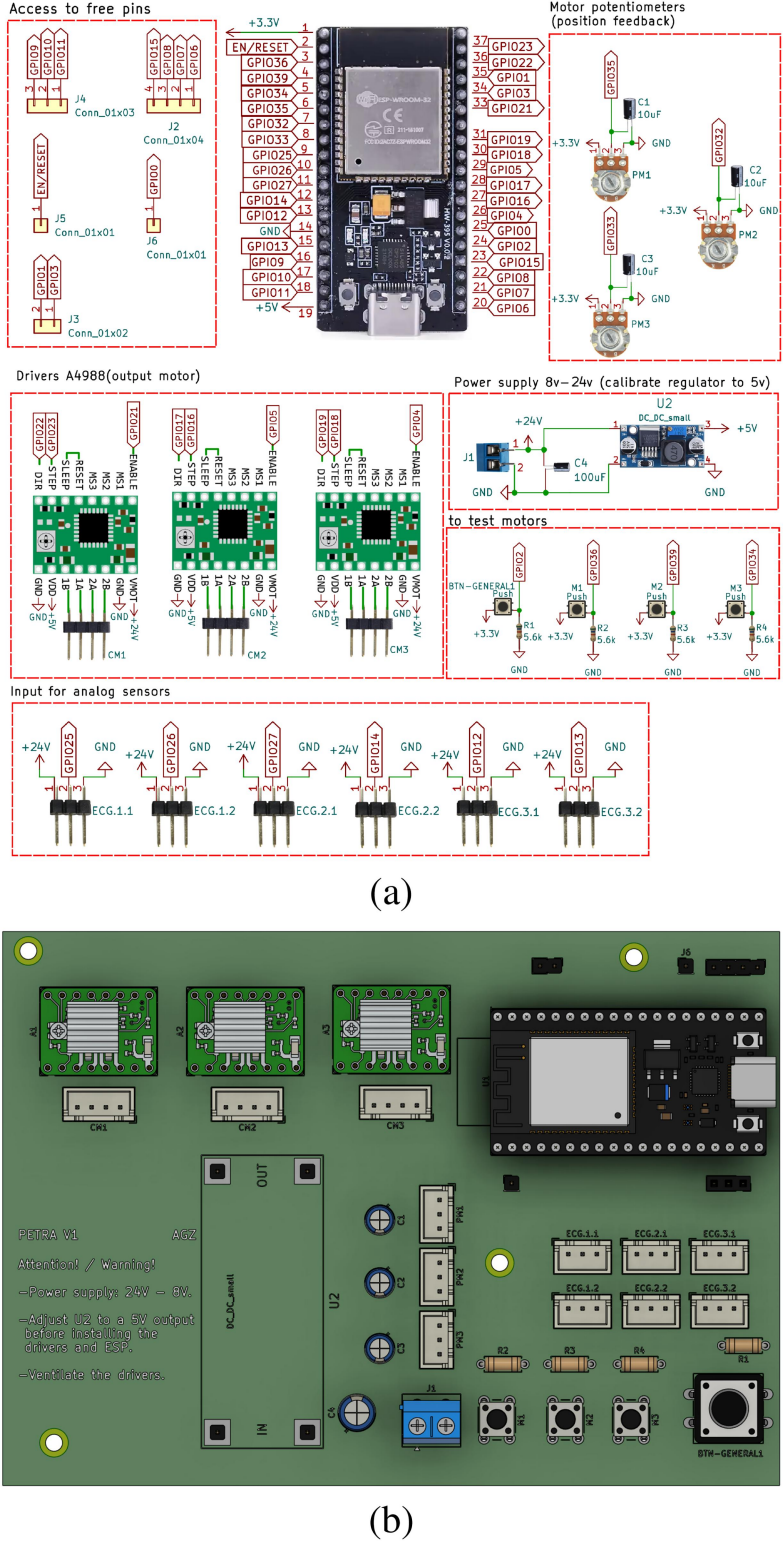
Electronic architecture and hardware integration of the PETRA system: **(a)** Schematic diagram with functional block segmentation for power management and signal conditioning, **(b)** Technical visualization (render) of the custom PCB. The design prioritizes safe operation and hardware integrity through the use of informative voltage silkscreening, physical isolation of power planes, and a modular layout that optimizes controller ventilation and system maintainability.

The electronic architecture centers on a custom PCB designed to provide a robust platform for the kinematic validation of the system. The design prioritizes signal integrity through the physical isolation between power planes and sensitive analog traces, minimizing electromagnetic interference (EMI) on the ADC feedback lines. This stability is vital for the discrete post-movement correction scheme. Signal conditioning is further enhanced by individual 10 μF bypass capacitors on each ADC line (Figure 6a), which act as passive low-pass filters to eliminate high-frequency noise without introducing perceptible latency. A critical metrological constraint of this architecture is the 12-bit resolution of the ESP32 ADC, which introduces a theoretical quantization error of approximately $0.07^{\circ }$–$0.09^{\circ }$ per LSB (31). This value represents the lower bound of the system’s measurable error and must be considered when interpreting positioning accuracy.

The final PCB design (Figure 6b) was optimized for manufacturing through mechanical milling, ensuring high dimensional repeatability for seamless integration within the robot’s hexagonal base chassis. Additionally, the board provides modular ports for the future integration of physiological sensors such as EMG or FSR. This foresight allows for evolution toward impedance or myoelectric control strategies without hardware modifications, consolidating PETRA as a versatile research station.

The power chain drives the NEMA 17 motors (30) through A4988 controllers (32), utilizing a stabilized supply to protect the ESP32 and drivers against power fluctuations. A key feature for the system’s scalability is the inclusion of six 3-pin auxiliary ports for the future integration of physiological sensors, such as EMG or FSR. This modular design allows for the implementation of impedance or myoelectric control strategies without requiring physical modifications to the motherboard, consolidating PETRA as a versatile research station.

### 4.2 Firmware

**Figure 7:**
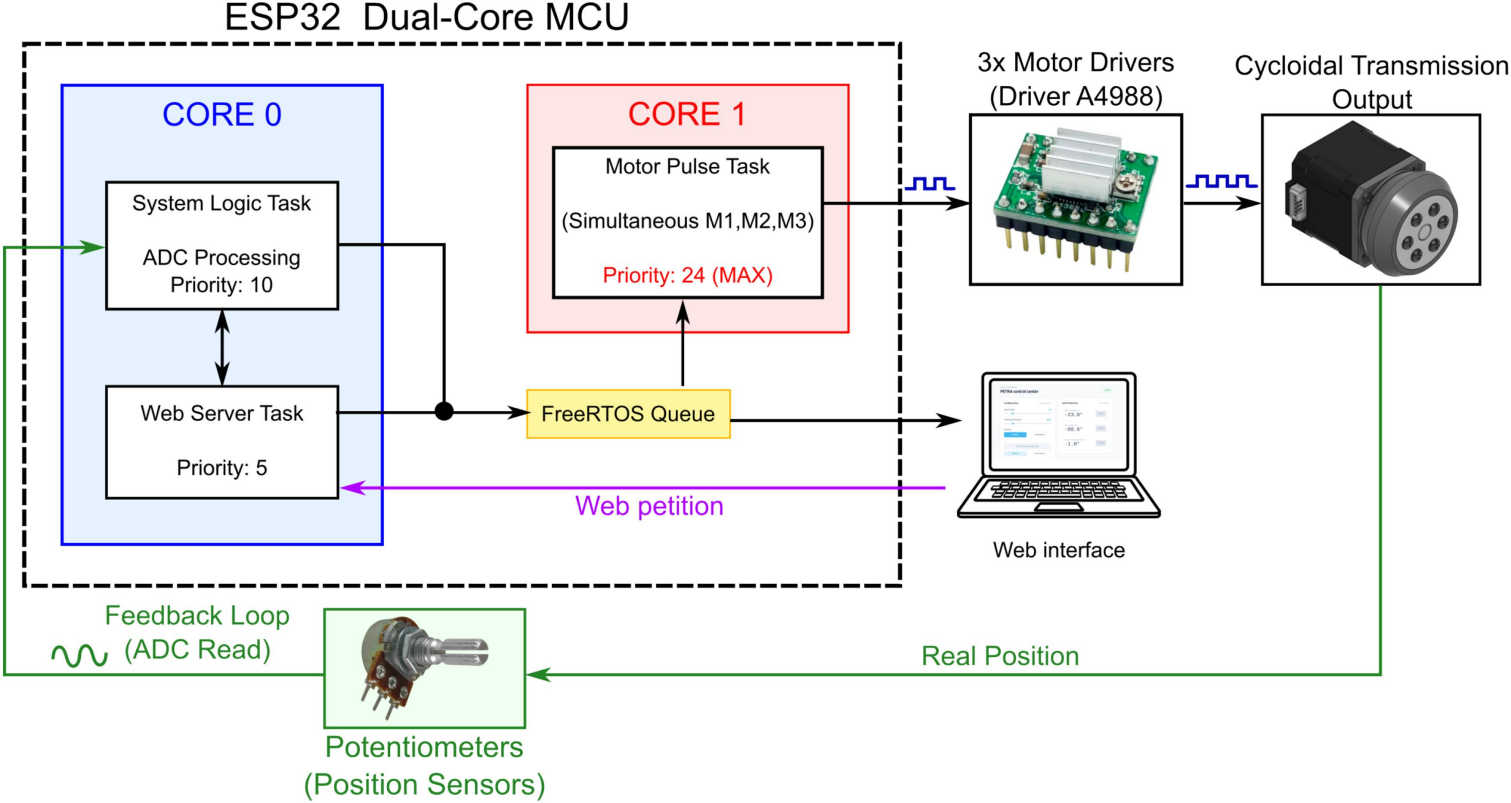
Dual-core firmware architecture: Core 1 dedicated to pulse generation (priority 24), Core 0 to communication and logic, synchronized via FreeRTOS queue.

The PETRA firmware was developed entirely under the ESP-IDF framework, selected for the granular control it offers over core affinity and priority management in FreeRTOS (31, 33). As illustrated in Figure 7, the system architecture deterministically decouples critical pulse generation from wireless communication through a dual-execution strategy:

**Core 1** (Actuation) executes the motorPulseTask with maximum priority (24). This isolation ensures that frequency generation for the three simultaneous motors is immune to network interrupts or file system latencies, ensuring fluid movement without temporal fluctuations (jitter).

**Core 0** (Application and Network): Manages the higher-level control logic and the HTTP server with lower priorities. Communication with the actuation core is carried out through a FreeRTOS queue (Figure 7), which acts as an asynchronous buffer, eliminating any temporal dependency between the communication layer and the physical execution layer (33).

**Figure 8:**
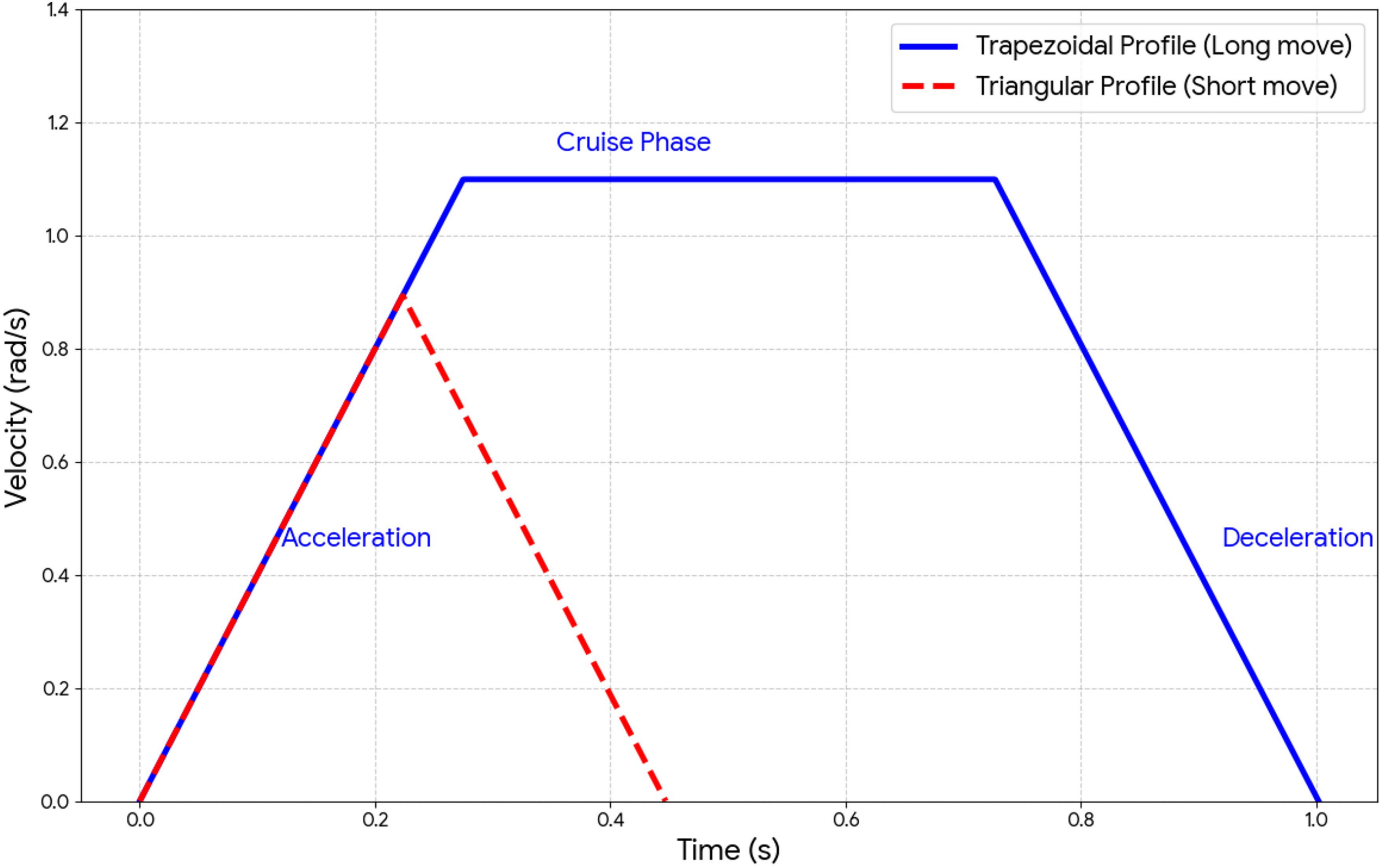
Embedded trapezoidal velocity profile.

The core contribution of the firmware lies in its embedded trapezoidal trajectory planner (Figure 8), which grants full autonomy to the system by eliminating the dependence on external controllers, ROS, or dedicated workstations (30, 33). The planner operates in the discrete step space, where the angular size of a step is defined as:

$$\alpha =\frac{2\pi }{\text{Steps Per Rev}}$$ (1)

From this parameter, the system dynamically calculates the number of steps required for the acceleration and deceleration phases, as defined in Equation 2:

$$N_{\text{accel}}=\frac{V_{\text{target}}^{2}}{2\times a_{\text{max}}\times \alpha }$$ (2)

As observed in the comparative analysis in Figure 8, if the value of $N_{\text{accel}}$ exceeds half of the total travel, the profile automatically degenerates into a triangular geometry. This real-time adjustment ensures smooth velocity transitions even in short displacements, eliminating mechanical impacts without the need for additional external logic (30). The interval between pulses is updated at each step through the relationship:

$$\text{Delay}\text{us}=\left(\frac{\alpha }{Y\text{actual}}\right)\times 10^{6}$$ (3)

This method results in continuous and deterministic velocity modulation. The three axes execute these profiles simultaneously according to the velocity, acceleration, and joint limit parameters detailed in Table 2, replicating the cadence of human therapeutic movement with an operational limit of 15 RPM on the output shaft to ensure the clinical representativeness of the trial (27).

**Table 2:** Velocity, acceleration, and joint limit parameters.

Motor	Max. Velocity	Max. Accel	Limits
BACK—ESPALDA (M1)	0.8 rad/s	4.0 rad/$s^{2}$	$0^{\circ }$/$+30^{\circ }$
ARM—BRAZO (M2)	0.8 rad/s	4.0 rad/$s^{2}$	$-135^{\circ }$/$+90^{\circ }$
FOREARM—ANTEBRAZO (M3)	1 rad/s	15.0 rad/$s^{2}$	$0^{\circ }$/$+135^{\circ }$

Limit validation operates on two levels: the HTTP handler rejects commands outside the ranges defined in Table 2 before queuing them, and the firmware blocks them again during execution by applying Equations 1, 3 to prevent mechanical failures. In the current architecture, these ROM limiters are purely electronic, acting as a software-defined safety layer. While mechanical hard stoppers were omitted in this prototype to facilitate rapid adjustments to the kinematic range during feasibility testing, their absence is recognized as a limitation for failsafe operation ; consequently, physical limiters are proposed as a priority hardware improvement for future versions to protect the system in the event of an electronic failure.

**Figure 9:**
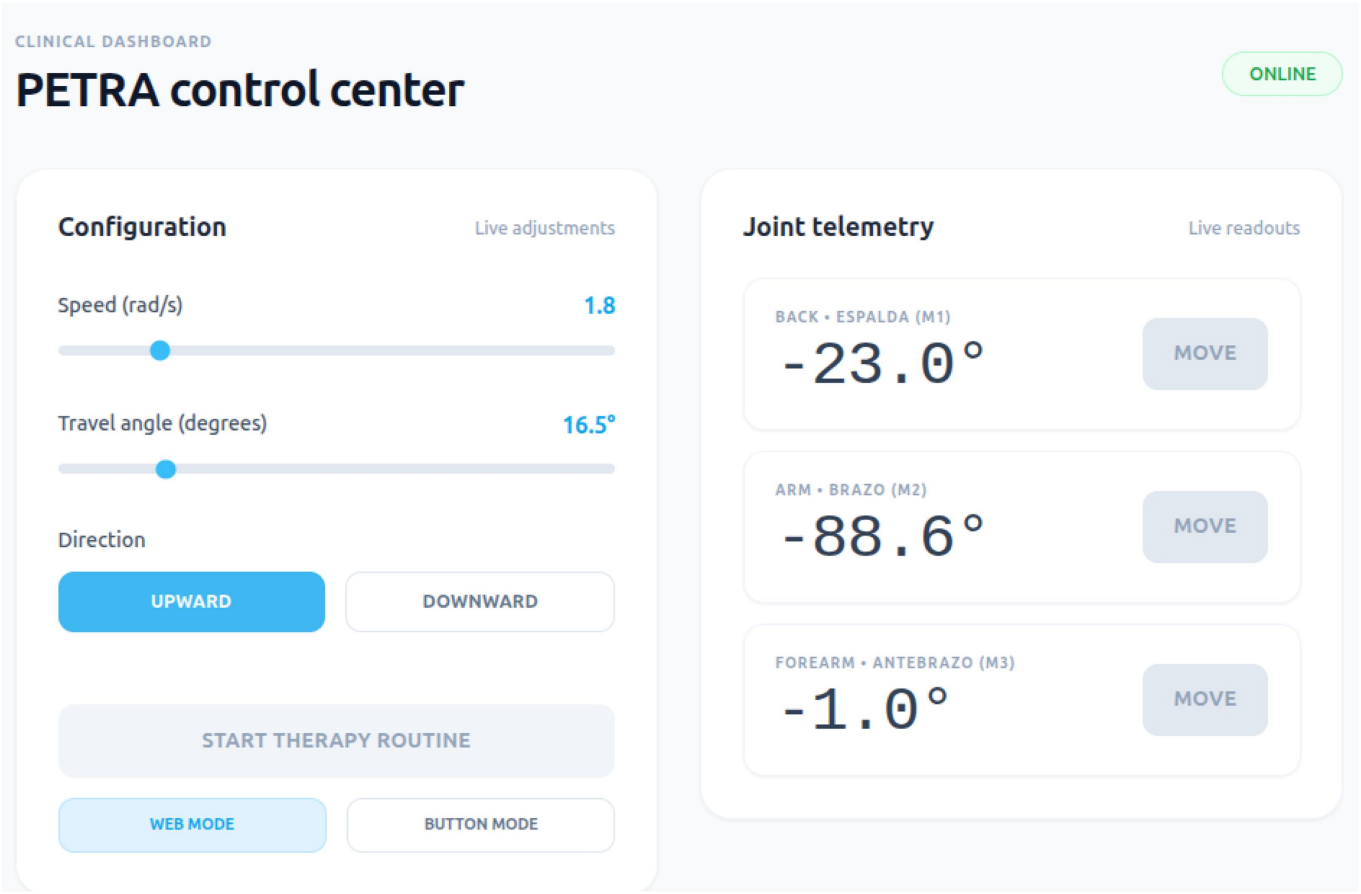
PETRA control panel: speed, angle, and direction configuration via sliders, real-time telemetry for the three axes, Web/Button Mode switching, and connection indicator.

The ESP32 operates as an autonomous WiFi access point serving the clinical control interface in Figure 9, which provides real-time telemetry of the three axes, dynamic speed and angle configuration via sliders, and a preprogrammed therapeutic routine. This routine executes the three axes in sequence with a discrete post-movement correction scheme that compares the target angle with the potentiometer reading, applying up to three correction cycles with a tolerance of $1.5^{\circ }$ before advancing to the next movement, guaranteeing the operational stability of the embedded system (33).

## 5 Test, validation and discussion

**Figure 10:**
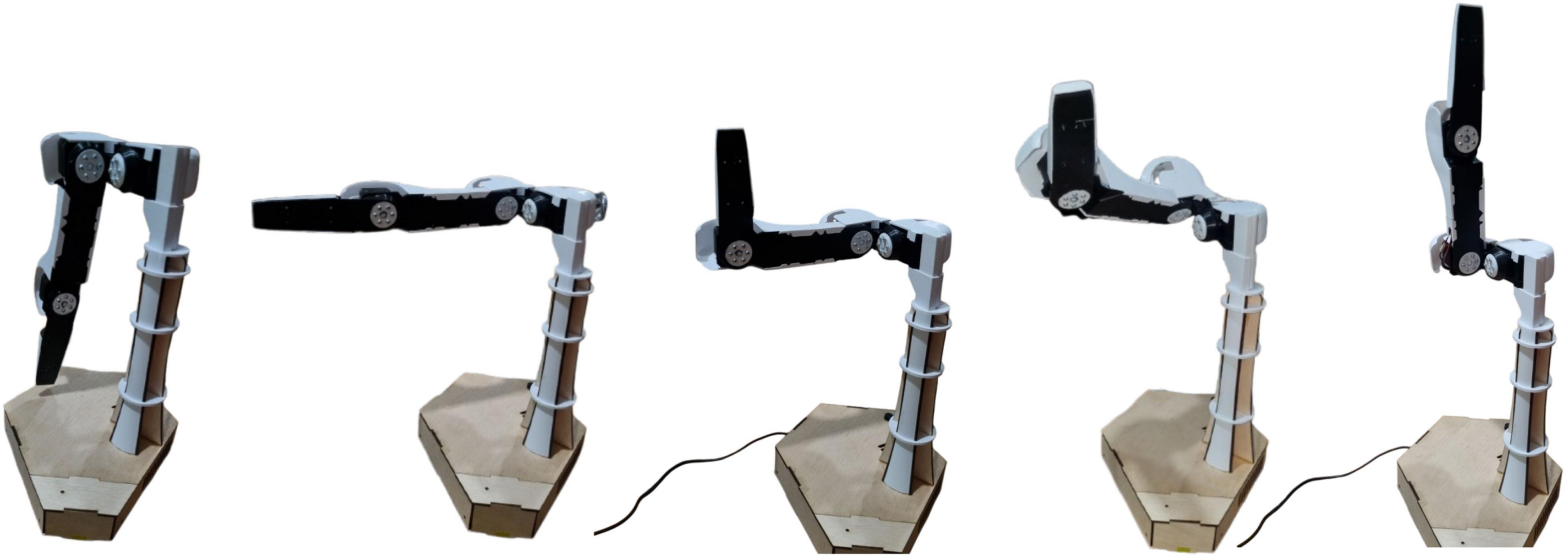
Physical prototype of PETRA in five different joint poses.

To contextualize the technical significance of PETRA, a quantitative comparison with prominent open-source and commercial platforms is presented in Table 3. While medical commercial exoskeletons typically exceed $10,000 and modular frameworks like OpenExo require high-cost commercial actuators, the total manufacturing cost of PETRA remains approximately $115. This budget covers three NEMA 17 stepper motors, ESP32-based control electronics, and roughly 200g of PETG filament, achieving a cost per joint under $25. Even when compared to the EULR system, which is restricted to functional hand rehabilitation, PETRA provides a robust 3-DOF testbed for proximal joints with a superior mechanical complexity-to-cost ratio. The physical prototype in different joint configurations is shown in Figure 10.

**Table 3:** Quantitative comparison between PETRA and existing platforms.

Feature	PETRA (This work)	EULR	OpenExo	Comm.
Total cost (USD)	≈115	98.40	>1,500${}^{a}$	>10,000
Cost/Axis (USD)	<25	N/A	High (Comm.)	>3,000
Actuated DOF	3 (Proximal)	Hand (Functional)	Modular	4–7
Architecture	Autonomous (ESP32)	Autonomous (ESP32)	Dependent (PC/ROS)	Proprietary
Transmission	Cycloidal (PETG)	Direct Drive	Conv. Gears	Harmonic
Open-Source	Yes	Yes	Yes	No

${}^{a}$Estimated cost based on high-end commercial actuators required for the framework.

It is worth noting that neither EULR nor OpenExo report pose accuracy or repeatability metrics under a standardized protocol such as ISO 9283, which precludes a direct numerical comparison of kinematic performance. The AP values obtained by PETRA—mean below $0.43^{\circ }$ and IQR within $\pm 1^{\circ }$ across all axes—are consistent with what would be expected from systems employing significantly more expensive commercial actuators, given that the dominant error source in PETRA is the dry friction of the bearingless PETG interfaces rather than the control architecture itself. This mechanical constraint, explicitly identified and bounded in this study, provides a clear engineering target for the next hardware iteration described in Section 6.

The experimental validation of PETRA follows the performance criteria defined in the ISO 9283 standard for industrial robots. To ensure methodological rigor, the system’s accuracy is characterized through Pose Accuracy (AP), representing the deviation between the commanded and reached positions, while its consistency is evaluated through Pose Repeatability (RP). A clinical tolerance of $\pm 3^{\circ }$ was adopted as a reference (19); however, this analysis transcends simple verification, seeking to characterize the fidelity of the PETG-manufactured cycloidal transmission and how mechanical efficiency conditions the dynamic behavior of the testbed.

The distribution of the 337 experimental trials is detailed in Table 4. The sample size was selected to ensure a standard error of the mean below 5% of the system’s theoretical resolution ($\approx 0.09^{\circ }$), which is fundamentally limited by the ADC resolution of the ESP32.

**Table 4:** Distribution of experimental trials per joint and target angle (n=337).

Motor	$15^{\circ }$	$30^{\circ }$	$45^{\circ }$	$60^{\circ }$	$90^{\circ }$	Total
M1 (Back)	28	28	–	–	–	56
M2 (Arm)	28	28	28	28	28	140
M3 (Forearm)	28	29	28	28	28	141
Total	84	85	56	56	56	337

Notably, although the hardware is mechanically capable of a $90^{\circ }$ range, motor M1 (Back) was intentionally restricted to $30^{\circ }$ via firmware. This software-defined safety limit was established to accommodate the natural biomechanics of the shoulder complex. Preliminary observations indicated that at higher angles, the lateral expansion of the torso and the abduction of the scapula, known as scapulohumeral rhythm (34), could lead to mechanical interference with the fixed backplate of the exoskeleton. To prevent user discomfort and ensure anatomical alignment during this feasibility stage, the range was capped to the safe functional zone for trunk-stabilized rehabilitation. The AP for each trial was processed using the metric defined in Equation 4:

$$AP_{q}=\theta_{\mathrm{target}}-\theta_{\mathrm{reached}}$$ (4)

**Figure 11:**
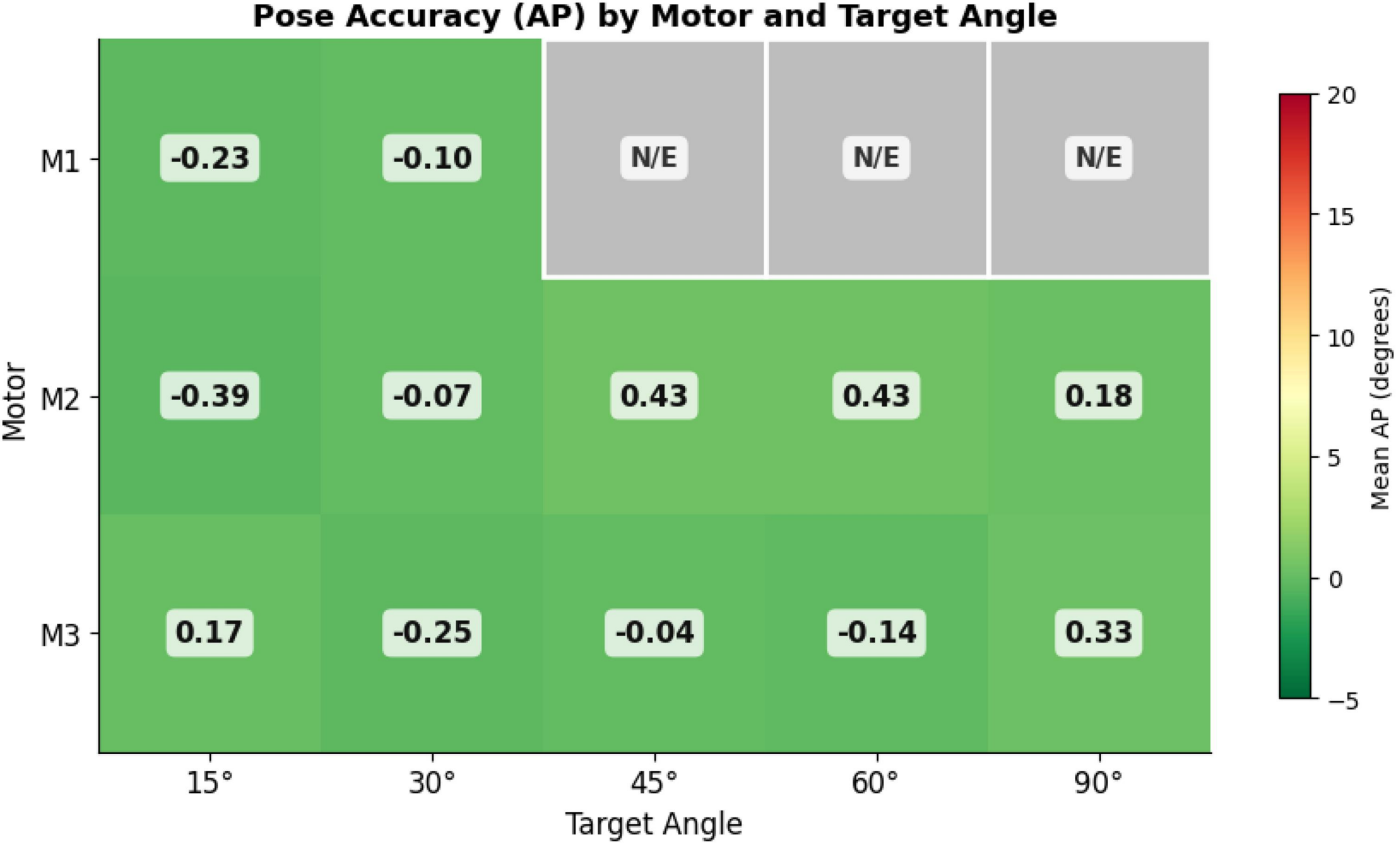
Spatial distribution of Pose Accuracy (AP) by motor and target angle. The gray area indicates anatomical safety restrictions for M1.

The results in the heatmap (Figure 11) reveal remarkable AP for an additive manufacturing system. M3 exhibits a mean AP of $-0.43^{\circ }$, attributed to the lower inertial load on the elbow axis. For M1 and M2, the AP remains at or below $0.43^{\circ }$ for all evaluated angles, suggesting that the cycloidal geometry effectively compensates for FDM printing tolerances.

To formally characterize the statistical properties of the positioning performance, a Wilcoxon signed-rank test (35) was applied to the AP measurements of each axis (performed on n=259 valid records after excluding records with data entry corruption), testing the null hypothesis that the population median AP equals zero. The test revealed a statistically significant negative systematic offset in all three axes: M1 (W=178.0, p<0.001), M2 (W=1700.5, p=0.001), and M3 (W=1165.0, p<0.001). The 95% confidence intervals for the mean AP were $\left[-0.79^{\circ },\;-0.31^{\circ }\right]$ for M1, $\left[-0.55^{\circ },\;-0.17^{\circ }\right]$ for M2, and $\left[-0.60^{\circ },\;-0.26^{\circ }\right]$ for M3. These results indicate a consistent but clinically negligible tendency to undershoot the commanded angle, likely attributable to the quantization floor of the ESP32 12-bit ADC (theoretical resolution ≈0.07–$0.09^{\circ }$ per LSB (31)) combined with the static friction of the PETG interfaces that opposes the final correction step. Critically, the magnitude of this bias remains well below the $\pm 3^{\circ }$ clinical tolerance established for upper limb rehabilitation (19), confirming that it does not compromise therapeutic applicability. The 95% confidence intervals for the limits of agreement (36) were $\left[0.61^{\circ },\;1.44^{\circ }\right]$ (upper) and $\left[-2.54^{\circ },\;-1.71^{\circ }\right]$ (lower) for M1, $\left[1.25^{\circ },\;1.92^{\circ }\right]$ (upper) and $\left[-2.63^{\circ },\;-1.97^{\circ }\right]$ (lower) for M2, and $\left[1.05^{\circ },\;1.64^{\circ }\right]$ (upper) and $\left[-2.50^{\circ },\;-1.91^{\circ }\right]$ (lower) for M3. All limits of agreement remain within the clinical tolerance band, demonstrating that command-feedback consistency is adequate for the intended application stage of PETRA.

**Figure 12:**
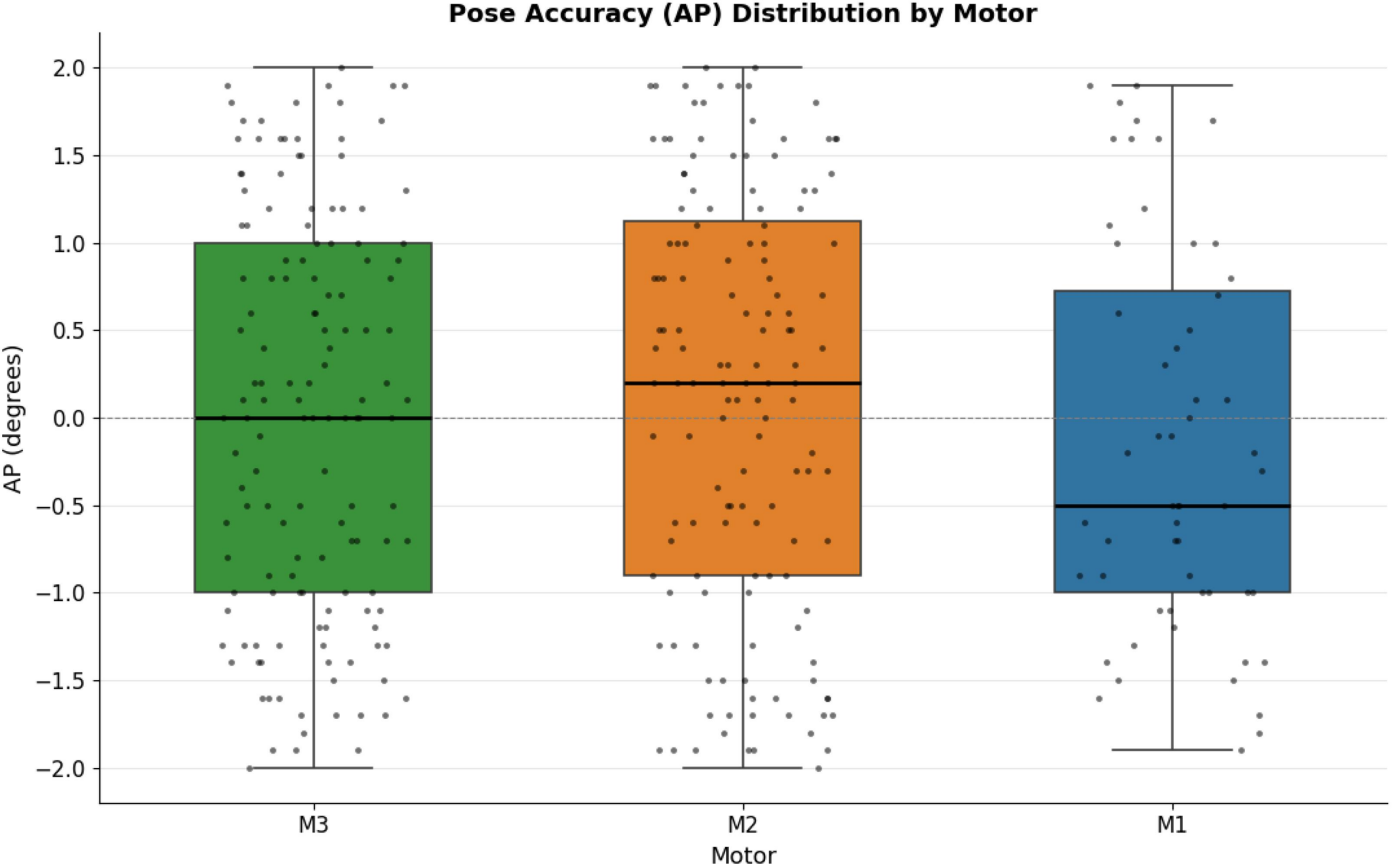
Pose Accuracy (AP) dispersion per motor. The consistency of the medians reflects the Pose Repeatability (RP) of the transmission.

The system’s consistency, or Pose Repeatability (RP), becomes evident in Figure 12. The box plots show that the interquartile range (IQR) for all three motors is contained within $\pm 1^{\circ }$, denoting high RP. The slight negative bias in M1 ($\approx -0.5^{\circ }$) indicates a systematic gain error, likely related to the analog resolution or structural deflection under the arm’s weight, which is correctable through software.

**Figure 13:**
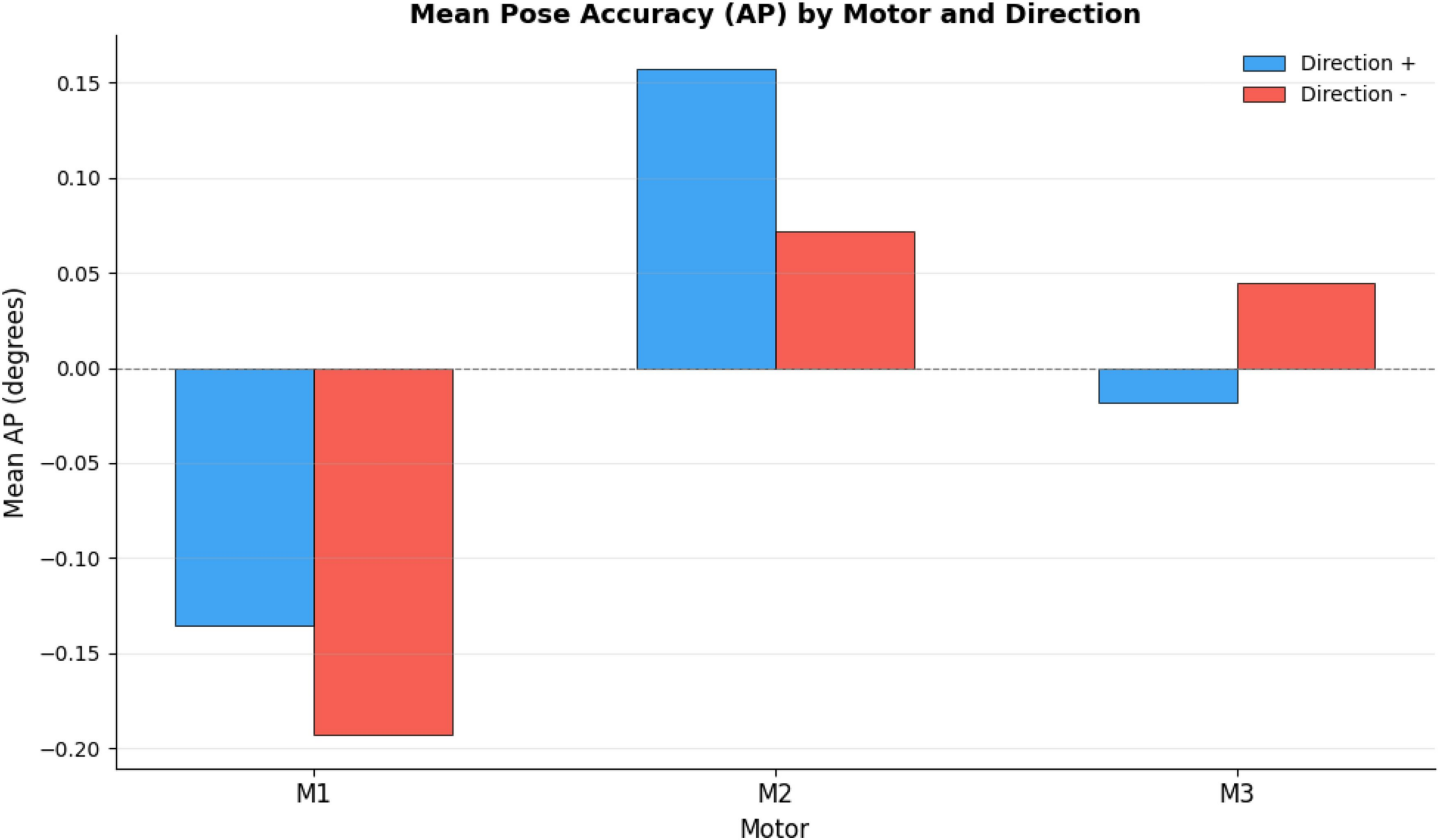
Comparison of AP according to rotation direction. Symmetry indicates the absence of significant backlash.

One of the greatest challenges in 3D-printed reducers is backlash. The bicyclic cycloidal architecture of PETRA mitigates this without costly industrial tolerances. As observed in Figure 13, the directional asymmetry in AP is minimal, not exceeding $0.16^{\circ }$ in M1 and M2; M3 exhibits a slightly higher global directional asymmetry of $0.26^{\circ }$, concentrated at the $30^{\circ }$ and $90^{\circ }$ target angles, which remains within the $\pm 3^{\circ }$ clinical tolerance (19) but warrants attention in future inter-session repeatability studies. This validates the design hypothesis: continuous contact of disk lobes with reaction pins eliminates dead zones typical of conventional gears (29).

**Figure 14:**
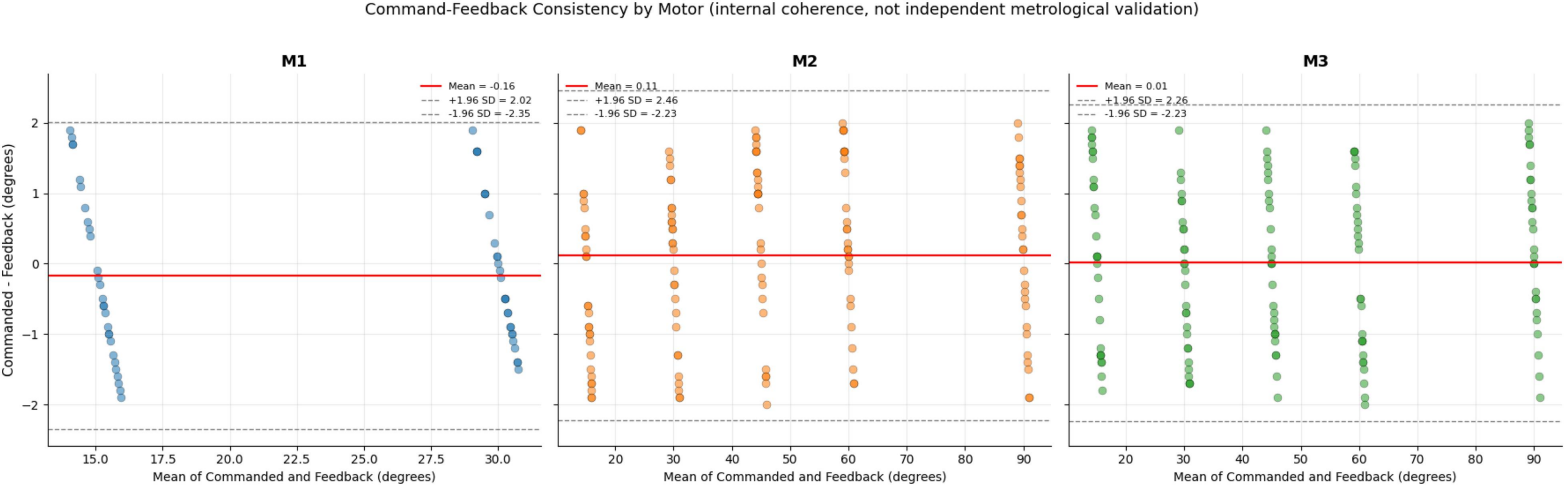
Bland-Altman plots by motor. The horizontal lines represent the 95% limits of agreement for command-feedback consistency.

The assessment of internal consistency was performed using a Bland-Altman analysis (Figure 14). However, as the feedback is obtained from internal potentiometers rather than an external gold-standard reference, this study formally describes these results as command-feedback consistency (36). This approach explicitly acknowledges the metrological circularity inherent in this prototyping stage while providing a robust verification of the mechanical integrity of the transmissions under load. With a global mean consistency of $0.01^{\circ }$ for M3 and $0.11^{\circ }$ for M2, the limits of agreement fall within $\pm 2.35^{\circ }$, placing PETRA within the clinical margins required for its intended application.

**Figure 15:**
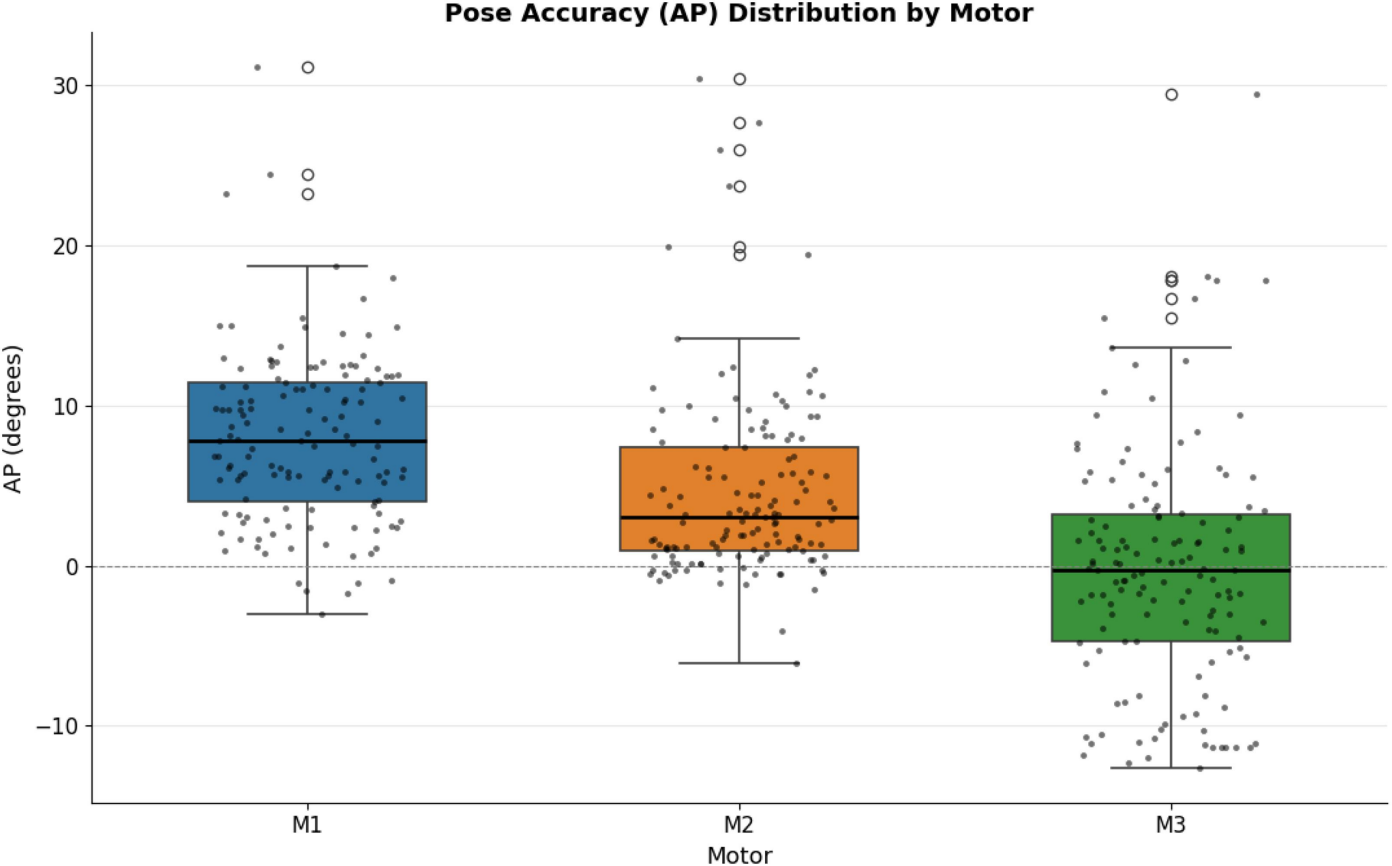
Accuracy degradation under high-speed regime. Note the drastic increase in the AP scale and the instability of M3.

Despite the excellent performance under standard conditions, the nature of PETG and the absence of metallic bearings impose a critical restriction: low efficiency due to dry friction. To explore this, the system was subjected to an increase in velocity seeking the dynamic torque saturation point. Figure 15 illustrates a radical change in the AP phenomenology. Under high-speed conditions, the M1 AP shifts towards $8^{\circ }$, while M2 presents a wider dispersion centered around $4^{\circ }$. This demonstrates that the torque required to overcome internal friction and load is close to the maximum capacity of NEMA 17 motors.

The most critical finding is observed in M3, where outliers reaching $90^{\circ }$ appear, representing a catastrophic loss of synchronism or stall. These results demonstrate that the viability of PETRA as a high-speed device depends on improving transmission efficiency, either through rolling elements or low-friction polymers. Therefore, the parameters in Table 2 represent the physical limit that guarantees the kinematic integrity of the exoskeleton in its current configuration.

## 6 Conclusion

The experimental validation of PETRA through 337 trials allowed for the characterization of the platform’s behavior as a functional 3-degree-of-freedom (DoF) exoskeletal testbed for kinematic validation in rehabilitation robotics (27). By following the performance criteria of the ISO 9283 standard, the study facilitated a precise quantification of Pose Accuracy (AP) and Pose Repeatability (RP), establishing a baseline for the development of replicable technical assistance protocols. A significant finding confirms negligible backlash in the bicyclic cycloidal transmission, where directional asymmetry in AP did not exceed $0.16^{\circ }$ in M1 and M2; the M3 axis showed a slightly higher global asymmetry of $0.26^{\circ }$, which nonetheless remains within the clinical tolerance band. This validates the effectiveness of the cycloidal geometry in eliminating mechanical dead zones, ensuring superior movement smoothness compared to conventional gears where mechanical hysteresis often degrades the integrity of the therapeutic trajectory.

In terms of operational precision under standard speeds, the system demonstrated performance consistent with clinical requirements. The elbow axis (M3) maintained a mean AP of $-0.43^{\circ }$ under standard operating conditions, while the proximal axes (M1 and M2) operated below $0.4^{\circ }$, placing the platform well within the clinical tolerance of $\pm 3^{\circ }$ required for assisted upper limb rehabilitation (19). Furthermore, the assessment of command-feedback consistency through Bland-Altman analysis (36) verified the internal reliability of the mechanical transmissions, even when acknowledging the metrological circularity of using internal feedback. The evaluation under high-speed regimes identified friction in the bearingless PETG interfaces as the dominant operational limitation. Under these conditions, the degradation of AP evidenced that low mechanical efficiency consumes the residual dynamic torque of the stepper motors as the frequency increases, marking the current physical limit for stable kinematic execution.

The dual-core firmware architecture implemented within the ESP-IDF framework constitutes a fundamental pillar of the system’s autonomy, achieving deterministic axis coordination without dependence on external computers or PC-based control stacks (31). This computational strategy, integrated with a custom single-layer PCB and housed within a standalone hexagonal pedestal base, allows for the deployment of the system as a transportable and ready-to-use research station. With a manufacturing cost per joint under 25 USD, PETRA reduces the economic barriers of commercial modular platforms by an order of magnitude, positioning itself as a viable alternative for institutions with limited resources. The open-source publication of design files reinforces this commitment to accessibility, fostering global replicability and collaborative advancement in affordable rehabilitation robotics (33).

Future development follows a two-track roadmap addressing the system’s primary mechanical and computational limitations identified in this study.

On the mechanical track, the dominant constraint identified experimentally is the high dry friction of the PETG-on-PETG and PETG-on-bushing interfaces, which consumes the available output torque and prevents the current architecture from delivering representative assistive forces to the user’s limb. The next hardware iteration will address this directly by redesigning the cycloidal transmission to incorporate metallic bushings, rolling-element bearings, and precision shoulder bolts at all rotating interfaces. This upgrade is expected to reduce friction losses substantially, allowing the transmission to deliver the full theoretical output torque of the NEMA 17 actuators and enabling, for the first time, meaningful force-interaction experiments with a physical load representing the user’s arm weight.

On the computational track, the current WiFi-based interface, while sufficient for laboratory feasibility testing, limits the system’s capacity for real-time trajectory planning and workspace computation. To address this, PETRA will adopt a master–slave architecture in which a Raspberry Pi 5 (37) acts as the high-level supervisor, handling trajectory generation, workspace management, and advanced control algorithms, while the ESP32 retains its role as the dedicated real-time actuator controller. The existing ESP-IDF/FreeRTOS firmware on the ESP32 will be preserved without modification to its core pulse generation and trapezoidal planning logic; only the command interface will be updated to receive motion commands from the Raspberry Pi via UART rather than from the web interface. This architecture preserves the deterministic real-time guarantees of the ESP32 while offloading computationally intensive tasks—such as inverse kinematics, wrench estimation, and supervisory safety monitoring—to the Raspberry Pi 5’s quad-core processor.

These two upgrades are interdependent: the mechanical improvement provides the actuator authority required to execute the trajectories that the enhanced computational layer will plan. Together, they constitute the foundation for transitioning PETRA from a kinematic validation testbed into a platform capable of force-interactive rehabilitation research, subject to institutional ethical approval prior to any contact with human subjects.

## Data Availability

The raw data supporting the conclusions of this article will be made available by the authors, without undue reservation.

## Footnotes

^1^ https://github.com/RAMEL-ESPOL/PETRA.git
